# Approaches for establishing the function of regulatory genetic variants involved in disease

**DOI:** 10.1186/s13073-014-0092-4

**Published:** 2014-10-31

**Authors:** Julian Charles Knight

**Affiliations:** Wellcome Trust Centre for Human Genetics, University of Oxford, Roosevelt Drive, Oxford, OX3 7BN UK

## Abstract

The diversity of regulatory genetic variants and their mechanisms of action reflect the complexity and context-specificity of gene regulation. Regulatory variants are important in human disease and defining such variants and establishing mechanism is crucial to the interpretation of disease-association studies. This review describes approaches for identifying and functionally characterizing regulatory variants, illustrated using examples from common diseases. Insights from recent advances in resolving the functional epigenomic regulatory landscape in which variants act are highlighted, showing how this has enabled functional annotation of variants and the generation of hypotheses about mechanism of action. The utility of quantitative trait mapping at the transcript, protein and metabolite level to define association of specific genes with particular variants and further inform disease associations are reviewed. Establishing mechanism of action is an essential step in resolving functional regulatory variants, and this review describes how this is being facilitated by new methods for analyzing allele-specific expression, mapping chromatin interactions and advances in genome editing. Finally, integrative approaches are discussed together with examples highlighting how defining the mechanism of action of regulatory variants and identifying specific modulated genes can maximize the translational utility of genome-wide association studies to understand the pathogenesis of diseases and discover new drug targets or opportunities to repurpose existing drugs to treat them.

## Introduction

Regulatory genetic variation is important in human disease. The application of genome-wide association studies (GWAS) to common multifactorial human traits has revealed that most associations arise in non-coding DNA and implicate regulatory variants that modulate gene expression [1]. Gene expression occurs in a dynamic functional epigenomic landscape in which the majority of genomic sequence is proposed to have regulatory potential [2]. Inter-individual variation in gene expression has been found to be heritable and can be mapped as quantitative trait loci (QTLs) [3,4]. Such mapping studies reveal that genetic associations with gene expression are common, that they often have large effect sizes, and that regulatory variants act locally and at a distance to modulate a range of regulatory epigenetic processes, often in a highly context-specific manner [5]. Indeed, the mode of action of such regulatory variants is very diverse, reflecting the complexity of mechanisms regulating gene expression and their modulation by environmental factors at the cell, tissue or whole-organism level.

Identifying regulatory variants and establishing their function is of significant current research interest as we seek to use GWAS for drug discovery and clinical benefit [6,7]. GWAS have identified pathways and molecules that were not previously thought to be involved in disease processes and that are potential therapeutic targets [8,9]. However, for the majority of associations, the identity of the genes involved and their mechanism of action remain unknown, which limits the utility of GWAS. An integrated approach is needed, taking advantage of new genomic tools to understand the chromatin landscape, interactions and allele-specific events, and reveal detailed molecular mechanisms.

Here I review approaches to understanding regulatory variation, from the viewpoint of both researchers needing to identify and establish the function of variants underlying a particular disease association, and those seeking to define the extent of regulatory variants and their mechanism of action at a genome-wide scale. I describe the importance of understanding context-specificity in resolving regulatory variants, including defining the disease-relevant epigenomic landscape in which variants operate, to enable functional annotation. I highlight the utility of eQTL studies for linking variants with altered expression of genes and the experimental approaches for establishing function, including descriptions of recent techniques that can help. I provide a strategic view, illustrated by examples from human disease, that is relevant to variants occurring at any genomic location, whether in classical enhancer elements or other locations where there is the potential to modulate gene regulation.

## Regulatory variants and gene expression

Regulatory variation most commonly involves single-nucleotide variants (SNVs) but also encompasses a range of larger structural genomic variants that can affect gene expression, including copy number variation [10]. Gene regulation is a dynamic, combinatorial process involving a variety of elements and mechanisms that may only operate in particular cell types, at a given stage in development or in response to environmental factors [11,12]. Various events that are critical to gene expression are modulated by genetic variation: transcription factor binding affinity at enhancer or promoter elements; disruption of chromatin interactions; the action of microRNAs or chromatin regulators; alternative splicing; and post-translational modifications [13,14]. Classical epigenetic marks such as DNA methylation, chromatin state or accessibility can be modulated directly or indirectly by variants [15-18]. Changes in transcription factor binding related to sequence variants are thought to be a principal driver of changes in histone modifications, enhancer choice and gene expression [17-19].

Functional variants can occur at both genic and intergenic sites, with consequences that include both up- and down-regulation of expression, differences in the kinetics of response or altered specificity. The effect of regulatory variants depends on the sequences that they modulate (for example, promoter or enhancer elements, or encoded regulatory RNAs) and the functional regulatory epigenomic landscape in which they occur. This makes regulatory variants particularly challenging to resolve, as this landscape is typically dynamic and context specific. Defining which sequences are modulated by variants has been facilitated by several approaches: analysis of signatures of evolutionary selection and sequence conservation; experimental identification of regulatory elements; and epigenomic profiling in model organisms, and more recently in humans, for diverse cell and tissue types and conditions [15,20].

The understanding of the consequences of genetic variation for gene expression provides a more tractable intermediate molecular phenotype than a whole-organism phenotype, where confounding by other factors increases heterogeneity. This more direct relationship with underlying genetic diversity might account in part for the success of approaches resolving association with transcription of sequence variants, such as eQTL mapping [3,5].

## Regulatory variants, function and human disease

The heritable contribution to common polygenic disease remains challenging to resolve, but GWAS have now mapped many loci with high statistical confidence. Over 90% of trait-associated variants are found to be located in non-coding DNA, and they are significantly enriched in chromatin regulatory features, notably DNase I hypersensitive sites [21]. Moreover, there is significant overrepresentation of GWAS variants in eQTL studies, implicating regulatory variants in a broad spectrum of common diseases [7].

Several studies have identified functional variants involving enhancer elements and altered transcription factor binding. These include a GWAS variant associated with renal cell carcinoma that results in impaired binding and function of hypoxia inducible factor at a novel enhancer of *CCND1* [22]; a common variant associated with fetal hemoglobin levels in an erythroid-specific enhancer [23]; and germline variants associated with prostate and colorectal cancer that modulate transcription factor binding at enhancer elements involving looping and long-range interactions with *SOX9* [24] and *MYC* [25], respectively. Multiple variants in strong linkage disequilibrium (LD) identified by GWAS can exert functional effects through various different enhancers, resulting in cooperative effects on gene expression [26].

Functional variants in promoters have also been identified that are associated with disease. These include the extreme situation in which a gain-of-function regulatory SNV created a new promoter-like element that recruits GATA1 and interferes with expression of downstream α-globin-like genes, resulting in α-thalassemia [27]. Other examples include a Crohn’s-disease-associated variant in the 3’ untranslated region of *IRGM* that alters binding by the microRNA mir-196, enhancing mRNA transcript stability and altering the efficacy of autophagy, thus affecting the anti-bacterial activity of intestinal epithelial cells [28]. Some SNVs show significant association with differences in alternative splicing [29], which may be important for disease, as illustrated by a variant of *TNFRSF1A* associated with multiple sclerosis, which encodes a novel form of TNFR1 that can block tumor necrosis factor [30]. Disease-associated SNVs can also modulate DNA methylation resulting in gene silencing, as illustrated by a variant in a CpG island associated with increased methylation of the *HNF1B* promoter [31].

To identify functional variants, fine mapping of GWAS signals is vital. This can be achieved by using large sample sizes, incorporating imputation or sequence-level information, and involving diverse populations to maximize statistical confidence and resolve LD structure. Interrogation of available functional genomic datasets to enable functional annotation of identified variants and association with genes based on eQTL mapping is an important early step in prioritization and hypothesis generation. However, such analysis must take note of what is known of the pathophysiology of the disease, because the most appropriate cell or tissue type needs to be considered given the context-specificity of gene regulation and functional variants. Two case studies (Box 1) illustrate many of the different approaches that can be used to investigate the role of regulatory variants in loci identified by GWAS. These provide context for a more detailed discussion of techniques and approaches in the remainder of this review.

## Mapping regulatory variation

This section describes approaches and tools for functional annotation of variants, considering in particular the usefulness of resolving the context-specific regulatory epigenomic landscape and of mapping gene expression as a quantitative trait of transcription, protein or metabolites.

### Functional annotation and the regulatory epigenomic landscape

High-resolution epigenomic profiling at genome-wide scale using high-throughput sequencing (HTS) has enabled annotation of the regulatory landscape in which genetic variants are found and may act. This includes mapping regulatory features based on:

- chromatin accessibility using DNase I hypersensitivity (DNase-seq) mapping [32,33] and post-translational histone modifications by chromatin immunoprecipitation combined with HTS (ChIP-seq) [34] that indicate the location of regulatory elements such as enhancers;
- chromatin conformation capture (3C), which can be scaled using HTS to enable mapping of genome-wide interactions for all loci (Hi-C) [35] or for selected target regions (Capture-C) [36];
- targeted arrays or genome-wide HTS to define differential DNA methylation [15]; the non-coding transcriptome using RNA-seq to resolve short and long non-coding RNAs with diverse roles in gene regulation [37] that may be modulated by underlying genetic variation with consequences for common disease [38].

The ENCyclopedia Of DNA Elements (ENCODE) Project [2] has generated epigenomic maps for diverse human cell and tissue types, including chromatin state, transcriptional regulator binding and RNA transcripts, that have helped to identify and interpret functional DNA elements [20] and regulatory variants [1,39]. Enhancers, promoters, silencers, insulators and other regulatory elements can be context specific; this means that generating datasets for particular cellular states and conditions of activation of pathophysiological relevance will be necessary if we are to use such data to inform our understanding of disease. There is also a need to increase the amount of data generated from primary cells given the caveats inherent to immortalized or cancer cell lines. For example, although studies in lymphoblastoid cell lines (LCLs) have been highly informative [40], their immortalization using the Epstein-Barr virus may alter epigenetic regulation or specific human genes, notably DNA methylation, and observed levels of gene expression, affecting the interpretation of the effects of variants [41,42]. As part of ongoing efforts to expand the diversity of primary cell types and tissues for which epigenomic maps are available, the International Human Epigenome Consortium, which includes the NIH Roadmap Epigenetics Project [43] and BLUEPRINT [44], seeks to establish 1,000 reference epigenomes for diverse human cell types.

The FANTOM5 project (for ‘functional annotation of the mammalian genome 5’) has recently published work complementing and extending ENCODE by using cap analysis of gene expression (CAGE) and single-molecule sequencing to define comprehensive atlases of transcripts, transcription factors, promoters, enhancers and transcriptional regulatory networks [45,46]. This includes high-resolution context-specific maps of transcriptional start sites and their usage for 432 different primary cell types, 135 tissues and 241 cell lines, enabling promoter-level characterization of gene expression [46]. The enhancer atlas generated by FANTOM5 defines a map of active enhancers that are transcribed *in vivo* in diverse cell types and tissues [45]. It builds on the recognition that enhancers can initiate RNA polymerase II transcription to produce eRNAs (short, unspliced, nuclear non-polyadenylated non-coding RNAs) and act to regulate context-specific expression of protein-coding genes [45]. Enhancers defined by FANTOM5 were enriched for GWAS variants; the context specificity is exemplified by the fact that GWAS variants for Graves’ disease were enriched predominantly in enhancers expressed in thyroid tissue [45].

Publicly accessible data available through genome browsers significantly enhances the utility to investigators of ENCODE, FANTOM5 and other datasets that allow functional annotation and interpretation of regulatory variants, while tools integrating datasets in a searchable format further enable hypothesis generation and identification of putative regulatory variants (Table 1) [39,47,48]. The UCSC Genome Browser, for example, includes a Variant Annotation Integrator [49], and the Ensembl genome browser includes the Ensembl Variant Effect Predictor [50]. The searchable RegulomeDB database enables annotations for particular variants to be accessed. RegulomeDB combines data from ENCODE and other datasets, including manually curated genomic regions for which there is experimental evidence of functionality; chromatin state data; ChIP-seq data for regulatory factors; eQTL data; and computational prediction of transcription factor binding and motif disruption by variants [39]. Kircher and colleagues [47] recently published a Combined Annotation-Dependent Depletion method involving 63 types of genomic annotation to establish genome-wide likelihoods of deleteriousness for SNVs and small insertion-deletions (indels), which helps to prioritize functional variants.

**Table 1:** **Examples of online data resources and tools for analysis of putative regulatory variants**

**Name**	**Description**	**URL**
ENCODE	Encyclopedia of DNA Elements Project	https://www.encodeproject.org
FANTOM	Functional Annotation of the Mammalian Genome project	http://fantom.gsc.riken.jp/5/
International Human Epigenome Consortium	International Human Epigenome Consortium Data Portal	http://ihec-epigenomes.org/outcomes/ihec-data-portal/
Roadmap Epigenomics Project	NIH Roadmap Epigenomics Mapping Consortium, including links to data	http://www.roadmapepigenomics.org
BLUEPRINT	European hematopoietic epigenome project	http://www.blueprint-epigenome.eu
Variant Annotation Integrator (UCSC)	Tool for predicting functional effects of variants on transcripts	http://www.noncode.org/cgi-bin/hgVai
Variant Effect Predictor (Ensembl)	Integrated tool resolving effects of variant on regulatory regions, genes, transcripts and protein	http://www.ensembl.org/info/docs/tools/vep/index.html
RegulomeDB	Tool for functional annotation of SNVs including known and predicted regulatory elements and eQTLs	http://regulomedb.org
SNPnexus	Integrated functional annotation of SNVs	http://snp-nexus.org/about.html
JASPAR	Transcription factor binding profile database	http://jaspar.genereg.net
PROMO	Transcription factor binding site analysis	http://alggen.lsi.upc.es/cgi-bin/promo_v3/promo/promoinit.cgi?dirDB=TF_8.3
MAPPER2	Identification of transcription factor binding sites in multiple genomes	http://genome.ufl.edu/mapper/
HaploReg	Functional annotation of variants on haplotype blocks such as at GWAS loci	http://www.broadinstitute.org/mammals/haploreg/haploreg.php
GWAS3D	Integrated annotation of variants including chromatin interactions	http://jjwanglab.org/gwas3d
ORegAnno	Regulatory annotation database	http://www.oreganno.org/oregano/
ConSite	Transcription factor binding site detection using phylogenetic footprinting	http://consite.genereg.net
HGMD	Human Gene Mutation Database, including regulatory mutations	http://www.hgmd.org
Genevar	eQTL database integration, search and visualization	http://www.sanger.ac.uk/resources/software/genevar/
eQTL Browser	NCBI hosted browser to interrogate eQTL datasets	http://www.ncbi.nlm.nih.gov/projects/gap/eqtl/index.cgi
OMICStools	Links to a large number of multi-omics tools	http://omictools.com

eQTL, expression quantitative trait locus; GWAS, genome-wide association study; SNV, single-nucleotide variant.

Determining which variants are located in regulatory regions is further helped by analysis of conservation of DNA sequences across species (phylogenetic conservation) to define functional elements. Lunter and colleagues [51] recently reported that 8.2% of the human genome is subject to negative selection and is likely to be functional. Claussnitzer and colleagues [52] studied conservation of transcription factor binding sites in *cis*-regulatory modules. They found that the regulation involving such sequences was combinatorial and depended on complex patterns of co-occurring binding sites [52]. Application of their ‘phylogenic module complexity analysis’ approach to type 2 diabetes GWAS loci revealed a functional variant in the *PPARG* gene locus that altered binding of the homeodomain transcription factor PRRX1. This was experimentally validated using allele-specific approaches and effects on lipid metabolism and glucose homeostasis were demonstrated.

### Insights from transcriptome, proteome, and metabolome QTLs

**Figure 1:**
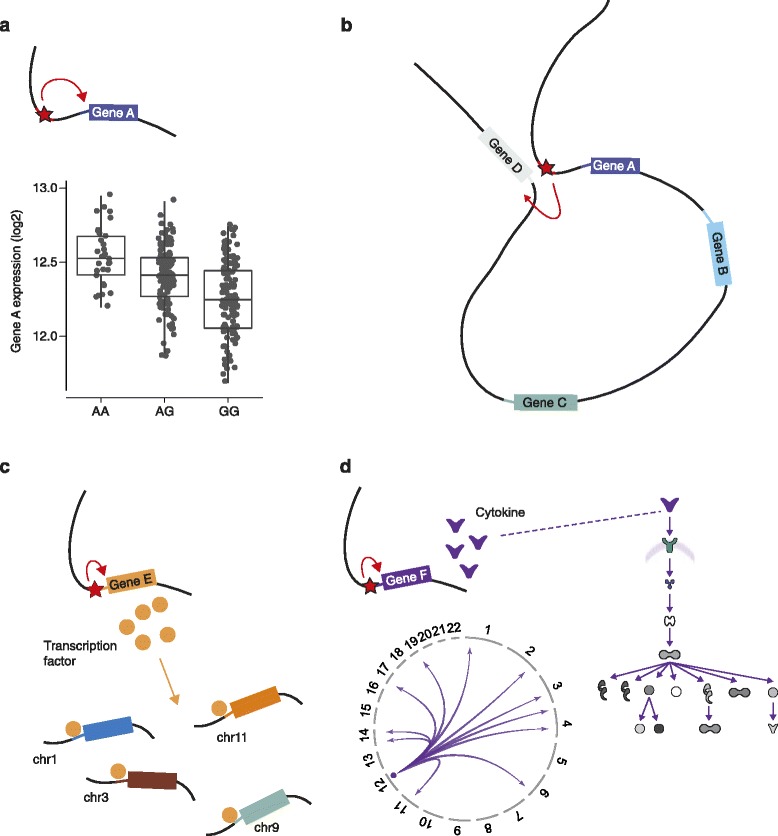
**Examples of local and distant effects of regulatory variants. (a)** A local *cis*-acting variant (red star, top) in a regulatory element (red line) affects allele-specific transcription factor binding affinity and is associated with differential expression of gene A (as shown by the chart, bottom), with possession of a copy of the A allele associated with higher expression than the G allele (hence AA homozygotes having higher expression than AG heterozygotes, with lowest expression in GG homozygotes). **(b)** The same variant can modulate expression of gene D at a distance through DNA looping that brings the regulatory enhancer element close to the promoter of gene D (gray line) on the same chromosome. **(c)** An example of a local *cis*-acting variant modulating expression of a transcription factor encoding gene, Gene E, differential expression of which modulates a set of target genes. Expression of these target genes is found to be associated in *trans* with the variant upstream of gene E. **(d)** A local *cis*-acting variant on chromosome 12 modulates expression of a cytokine gene and is also associated in *trans* with a set of genes whose expression is regulated through a signaling cascade determined by that cytokine. Such *trans* associations can be shown on a circos plot (chromosomes labeled 1-22 with arrows pointing to location of gene on a given chromosome).

Mapping gene expression as a quantitative trait is a powerful way to define the regions and markers associated with differential expression between individuals [53]. Application in human populations has enabled insights into the genomic landscape of regulatory variants, generating maps that are useful for GWAS, sequencing studies and other settings where the function of genetic variants is sought [5,7,54]. Local variants are likely to be *cis*-acting and those at a distance are likely to be *trans*-acting. Resolution of *trans*-eQTLs is challenging, requiring large sample sizes owing to the number of comparisons performed, because all genotyped variants in the genome can be considered for association. However, this resolution is important given how informative eQTLs can be for defining networks, pathways and disease mechanism [55]. When combined with *cis*-eQTL mapping, *trans*-eQTL analysis allows discovery of previously unappreciated relationships between genes, as a variant showing local *cis* association with expression of a gene might also be found to show *trans* association with one or more other genes (Figure 1). For example, in the case of a *cis*-eQTL involving a transcription factor gene, these *trans*-associated genes might be regulated by that transcription factor (Figure 1c). This can be very informative when investigating loci found in GWAS; for example, a *cis*-eQTL for the transcription factor KLF14 that is also associated with type 2 diabetes and high-density lipoprotein cholesterol was found to act as a master *trans* regulator of adipose gene expression [56]. *Trans*-eQTL analysis is also a complementary method to ChIP-seq for defining transcription factor target genes [57]. For other *cis*-eQTLs, the *trans*-associated genes might be part of a signaling cascade (Figure 1d), which might be well annotated (for example a *cis*-eQTL involving *IFNB1* is associated in *trans* with a downstream cytokine network) or provide new biological insights [57].

eQTLs are typically context specific, dependent for example on cell type [58-60] and state of cellular activation [57,61,62]. Careful consideration of relevant cell types and conditions is therefore needed when investigating regulatory variants for particular disease states. For example, eQTL analysis of the innate immune response transcriptome in monocytes defined associations involving canonical signaling pathways, key components of the inflammasome, downstream cytokines and receptors [57]. In many cases these were disease-associated variants and were identified only in induced monocytes, generating hypotheses for the mechanism of action of reported GWAS variants. Such variants would not have been resolved if only resting cells had been analyzed [57]. Other factors can also be significant modulators of observed eQTLs, including age, gender, population, geography and infection status, and they can provide important insights into gene-environment interactions [62-66].

The majority of published eQTL studies have quantified gene expression using microarrays. Application of RNA-seq enables high-resolution eQTL mapping, including association with abundance of alternatively spliced transcripts and quantification of allele-specific expression [40,67]. The latter provides a complementary mapping approach to define regulatory variants.

In theory, eQTLs defined at the transcript level might not be reflected at the protein level. However, recent work by Kruglyak and colleagues [68] in large, highly variable yeast populations using green fluorescent protein tags to quantify single-cell protein abundance has shown good correspondence between QTLs influencing mRNA and protein abundance; genomic hotspots were associated with variation in abundance of multiple proteins and modulating networks.

Mapping protein abundance as a quantitative trait (pQTL mapping) is important in ongoing efforts to understand regulatory variants and the functional follow-up of GWAS. However, a major limitation has been availability of appropriate high-throughput methods for quantification. A highly multiplexed proteomic platform involving modified aptamers was used to map *cis*-regulated protein expression in plasma [69], and micro-western and reverse-phase protein arrays enabled 414 proteins to be assayed simultaneously in LCLs, resolving a pQTL involved in the response to chemotherapeutic agents [70]. The application of state-of-the-art mass spectrometry-based proteomic methods is enabling quantification of protein abundance for pQTL mapping. There are still limitations, however, in the extent, sensitivity and dynamic range that can be assayed, the availability of analysis tools, and challenges inherent in studying the highly complex and diverse human proteome [71].

There are multiple ways in which genetic variation can modulate the nature, abundance and function of proteins, including effects of non-coding variants on transcription, regulation of translation and RNA editing, and alternative splicing. In coding sequences, non-synonymous variants can also affect regulation of splicing and transcript stability. An estimated 15% of codons have been proposed by Stergachis and colleagues [72] to specify both amino acids and transcription factor binding sites; they found evidence that the latter resulted in codon constraint through evolutionary selective pressure, and that coding SNVs directly affected the resultant transcription factor binding. It remains unclear to what extent sequence variants modulate functionally critical post-translational modifications, such as phosphorylation, glycosylation and sulfation.

The role of genetic variation in modulating human blood metabolites was highlighted by a recent large study by Shin and colleagues [73] of 7,824 individuals, in which 529 metabolites in plasma or serum were quantified using liquid-phase chromatography, gas chromatography and tandem mass spectrometry. This identified genome-wide associations at 145 loci. For specific genes, there was evidence of a spectrum of genetic variants ranging from very rare loss-of-function alleles leading to metabolic disorders to common variants associated with molecular intermediate traits and disease. Availability of eQTL data through gene expression profiling at the same time as metabolomic measurements enabled a Mendelian randomization analysis (a method for assessing causal associations in observational data that are based on the random assortment of genes from parents to offspring [74]) to search for a causal relationship between differential expression of a gene and metabolite levels using genetic variation as an instrumental variable. There were limitations due to study power but a causal role for some eQTLs in metabolic trait associations was defined, including for the acyl-CoA thioesterase *THEM4* and the cytochrome P450 *CYP3A5* genes [73].

Finally, analysis of epigenetic phenotypes as quantitative traits has proved very informative. Degner and colleagues [16] analyzed DNase-I hypersensitivity as a quantitative trait (dsQTLs) in LCLs. Many of the observed dsQTLs were found to overlap with known functional regions, show allele-specific transcription factor binding and also show evidence of being eQTLs. Methylation QTL (meQTL) studies have also been published for a variety of cell and tissue types that provide further insight in regulatory functions of genomic variants [75-77]. A meQTL study in LCLs revealed significant overlap with other epigenetic marks, including histone modifications and DNase-I hypersensitivity, and also with up- and down-regulation of gene expression [77]. Altered transcription factor binding by variants was found to be a key early step in the regulatory cascade that may result in altered methylation and other epigenetic phenomena [77].

## Methods for functional validation of variants

In this section I review different approaches and methodologies that can help establish mechanism for regulatory variants. These tools can be used to test hypotheses that have been generated from functional annotation of variants and eQTL mapping. In some instances, data will be publicly available through repositories or accessible through genome browsers to enable analysis (Table 1), for example in terms of allele-specific expression or chromatin interactions, but as previously noted the applicability and relevance of this information needs to be considered in the context of the particular variant and disease phenotype being considered. New data may need to be generated by the investigator. For both allele-specific gene expression and chromatin interactions, the new data can be analyzed in a locus-specific manner without the need for high-throughput genomic technologies, but equally it can be cost- and time-effective to screen many different loci simultaneously. A variety of other tools can be used to characterize variants, including analysis of protein-DNA interactions and reporter gene expression (Box 1). New genome editing techniques provide an exciting, tractable approach for studying human genetic variants, regulatory elements and genes in a native chromosomal context.

### Allele-specific transcription

*Cis*-acting regulatory variants modulate gene expression on the same chromosome. Resolution of allele-specific differences in transcription can be achieved using transcribed SNVs to establish the allelic origin of transcripts in individuals heterozygous for those variants [78]. Alternatively, it is possible to use proxies of transcriptional activity, such as phosphorylated RNA polymerase II (Pol II), to expand the number of informative SNVs, as these are not restricted to transcribed variants and can include any SNVs within about 1 kb of the gene when analyzed using allele-specific Pol II ChIP [79]. Early genome-wide studies of allele-specific expression showed that, in addition to the small number of classical imprinted genes showing monoallelic expression, up to 15 to 20% of autosomal genes show heritable allele-specific differences (typically 1.5- to 2-fold in magnitude), consistent with the widespread and significant modulation of gene expression by regulatory variants [80]. Mapping allele-specific differences in transcript abundance is an important complementary approach to eQTL mapping, as shown by recent high-resolution RNA-seq studies [40,81]. Lappalainen and colleagues [40] analyzed LCLs from 462 individuals from diverse populations in the 1000 Genomes Project. An integrated analysis showed that almost all the identified allele-specific differences in expression were driven by *cis*-regulatory variants rather than genotype-independent allele-specific epigenetic effects. Rare regulatory variants were found to account for the majority of identified allele-specific expression events [40]. Battle and colleagues [81] mapped allele-specific gene expression as a quantitative trait using RNA-seq in whole blood from 922 individuals, showing that this method is complementary to *cis*-eQTL mapping and can provide mechanistic evidence of regulatory variants acting in *cis*.

Allele-specific transcription factor recruitment provides further mechanistic evidence for how regulatory variants act. Genome-wide analyses - for example, of binding of the NF-κB transcription factor family by ChIP-seq [82] - have provided an overview of the extent of such events, but such datasets currently remain limited in terms of the numbers of individuals and transcription factors profiled. For some putative regulatory variants, predicting consequences for transcription factor binding by modeling using position-weighted matrices has proved powerful [83], and this can be improved using flexible transcription factor models based on hidden Markov models to represent transcription factor binding properties [84]. Experimental evidence for allele-specific differences in binding affinity can be generated using highly sensitive *in vitro* approaches such as electrophoretic mobility shift assays, while *ex vivo* approaches such as ChIP applied to heterozygous cell lines or individuals can provide direct evidence of relative occupancy by allele [85]. A further elegant approach is the use of allele-specific enhancer trap assays, successfully used by Bond and colleagues to identify a regulatory SNP in a functional p53 binding site [86].

### Chromatin interactions and DNA looping

**Figure 2:**
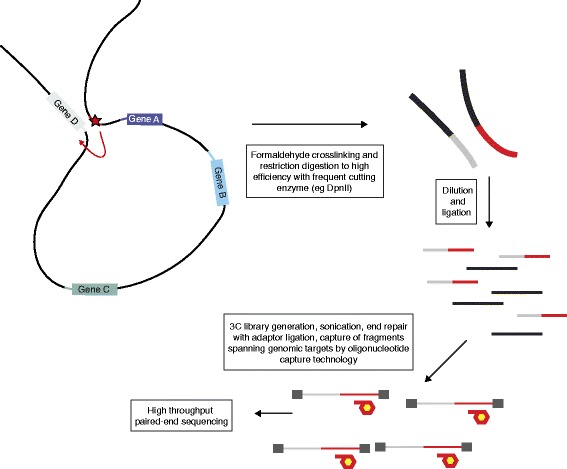
**Overview of the Capture-C approach.** Capture-C [36] enables mapping of chromatin interactions, in this example between a regulatory element (within the region denoted by a red line) and a gene promoter (gray line). Crosslinking and high-efficiency restriction digestion followed by proximity ligation (in which close proximity will favor ligation taking place, in this example generating red-gray lines in contrast to black lines representing other ligation events) allows such interactions to be defined. A 3C library is generated, sonicated and end repair performed with ligation of adaptors (dark gray boxes). Capture of target regions of interest (in this example target is region denoted by red line) involves oligonucleotide capture technology (capture probes denoted by red hexagons with yellow centers). Sequencing using end-ligated adapters allows genome-wide sites of interaction to be revealed. The approach can be multiplexed to several hundred targets.

Physical interactions between *cis*-regulatory elements and gene promoters can be identified by chromatin conformation capture methods, which provide mechanistic evidence to support hypotheses regarding the role of distal regulatory elements in modulating expression of particular genes and how this may be modulated by specific regulatory genetic variants. For some loci and target regions, 3C remains an informative approach, but typically investigators following up GWAS have several associated loci of interest to interrogate. Here, use of the Capture-C approach [36] (Figure 2) developed by Hughes and colleagues holds considerable promise: this high-throughput approach enables mapping of genome-wide interactions for several hundred target genomic regions spanning expression-associated variants and putative regulatory elements at high resolution. To complement and confirm those results it is also possible to analyze promoters of expression-associated genes as target regions. 3C methods can thus provide important mechanistic evidence linking GWAS variants to genes. Careful selection of the appropriate cellular and environmental context in which such variants act remains important, given that chromatin interactions are dynamic and context specific. Looping of chromatin can cause interaction between two genetic loci or epistatic effects, and there is evidence from gene expression studies that this is relatively common in epistatic networks involving common SNVs [87,88].

### Advances in genome editing techniques

**Figure 3:**
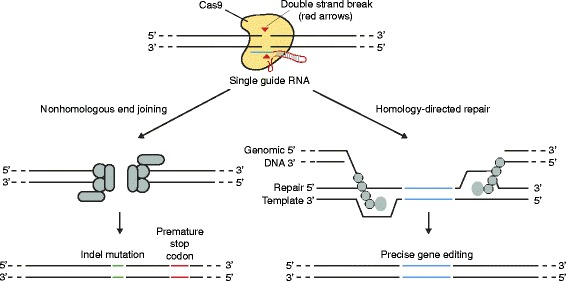
**Overview of the CRISPR-Cas9 system.** Cas-9 is a nuclease that makes a double-strand break at a location defined by a guide RNA [108]. The latter comprises a scaffold (red) and a 20-nucleotide guide sequence (blue) that pairs with the DNA target immediately upstream of a 5’-NGG motif (this motif varies depending on the exact bacterial species of origin of the CRISPR used). There are two main approaches that can be followed. (Left) Repair of the double-strand break by nonhomologous end joining can be used to knock out gene function though incorporation of random indels at junction sites, where these occur within coding exons, leading to frameshift mutations and premature stop codons. (Right) Homology-directed repair can enable precise genome editing through the use of dsDNA-targeting constructs flanking insertion sequences or single-stranded DNA oligonucleotides to introduce single-nucleotide changes. Adapted with permission from [108].

Model organisms have been very important in advancing our understanding of regulatory variants and modulated genes (Box 1). Analysis of variants and putative regulatory elements in an *in vivo* epigenomic regulatory landscape (the native chromosomal context) for human cell lines and primary cells is now more tractable following advances in genome editing technologies such as transcription activator-like effector nucleases (TALENs) [89] and in particular the RNA-guided ‘clustered regularly interspaced short palindromic repeats’ (CRISPR)-Cas nuclease system [90-92]. The latter approach uses guide sequences (programmable sequence-specific CRISPR RNA [93]) to direct cleavage by the non-specific Cas9 nuclease and generate double-strand breaks at target sites, and either nonhomologous end joining or homology-directed DNA repair using specific templates leads to the desired insertions, deletions or substitutions at target sites (Figure 3). The approach is highly specific, efficient, robust and can be multiplexed to enable simultaneous genome editing at multiple sites. Off-target effects can be minimized using a Cas9 nickase [92]. CRISPR-Cas9 has been successfully used for positive and negative selection screening in human cells using lentiviral delivery [94,95] and to demonstrate functionality for particular regulatory SNVs [52,61]. Lee and colleagues [61] discovered a context-specific eQTL of *SLFN5* and used CRISPR-Cas9 to demonstrate loss of inducibility by IFNβ on conversion from the heterozygous to homozygous (common allele) state in a human embryonic kidney cell line. Claussnitzer and colleagues [52] used CRISPR-Cas9 and other tools to characterize a type-2-diabetes-associated variant in the *PPARG2* gene; they replaced the endogenous risk allele in a human pre-adipocyte cell strain with the non-risk allele and showed increased expression of the transcript.

## Integrative approaches and translational utility

Genomics-led research has significant potential to enhance drug discovery and enable more targeted use of therapeutics by implicating particular genes and pathways [8,96]. This requires greater focus on target discovery, characterization and validation in academia combined with better integration with industry. Combining GWAS with eQTL analysis enables application of Mendelian randomization approaches to infer causality for molecular phenotypes [73,74]; this can enhance potential translational utility by indicating an intervention that could treat the disease. Gene sets arising from GWAS are significantly enriched for genes encoding known targets and associated drugs in the worldwide drug pipeline; mismatches between current therapeutic indications and GWAS traits are therefore opportunities for drug repurposing [97]. For example, Sanseau and colleagues [97] identified registered drugs or drugs in development that target *TNFSF11*, *IL27* and *ICOSLG* as potential repurposing opportunities for Crohn’s disease, given mismatches between GWAS associations with Crohn’s involving these genes and current drug indications. To maximize the potential of GWAS for therapeutics, and in particular for drug repurposing, it is important to have better resolution of the identity of genes modulated by GWAS variants so that associations can be established between genes and traits. When an existing drug is known to be effective in a given trait, it can then be considered for use in a further trait that shows association with the same target gene.

Two examples illustrate how knowledge of functional regulatory variants and association with specific traits can guide likely utility and application. Okada and colleagues [8] recently showed how an integrated bioinformatics pipeline, using data from functional annotation, *cis*-eQTL mapping, overlap with genes identified as causing rare Mendelian traits (here, primary immunodeficiency disorders) and molecular pathway enrichment analysis, could help prioritize and interpret results of GWAS for rheumatoid arthritis with a view to guiding drug discovery. Fugger and colleagues [30] identified a GWAS variant in the tumor necrosis factor receptor gene *TNFR1* that can mimic effects of TNF-blocking drugs. The functional variant was associated by GWAS with multiple sclerosis, but not with other autoimmune diseases, and mechanistically it was found to result in a novel soluble form of TNFR1 that can block TNF. The genetic data parallel clinical experience with anti-TNF therapy, which in general is highly effective in autoimmune disease but in multiple sclerosis can promote onset or exacerbations. This work shows how knowing the mechanism and spectrum of disease association across different traits can help in developing and using therapeutics.

## Conclusions and future directions

The quest for regulatory genetic variants remains challenging but is facilitated by a number of recent developments, notably in terms of functional annotation and tools for genome editing, mapping chromatin interactions and identifying QTLs involving different intermediate phenotypes such as gene expression at the transcript and protein level. Integrative genomic approaches will further enable such work by allowing investigators to effectively combine and interrogate complex and disparate genomic datasets [98,99]. A recurring theme across different approaches and datasets is the functional context specificity of many regulatory variants, requiring careful selection of experimental systems and of cell types and tissues. As our knowledge of the complexities of gene regulation expands, the diverse mechanisms of action of regulatory variants are being recognized. Resolving such variants is of intrinsic biological interest, and fundamental to current efforts to translate advances in genetic mapping of disease susceptibility into clinical utility and therapeutic application. Establishing mechanism and identifying specific modulated genes and pathways is therefore a priority. Fortunately, we increasingly have the tools for these purposes, both to characterize variants and study them in a high-throughput manner.

Key bottlenecks that need to be overcome include the generation of functional genomics data in a broad range of cell and tissue types relevant to disease (for other key issues that remain to be resolved see Box 2). Cell numbers can be limiting for some technologies, and a range of environmental contexts need to be considered. Moving to patient samples is challenging given heterogeneity related, for example, to stage of disease and therapy, but will be an essential component of further progress in this area. QTL mapping has proven highly informative but similarly requires large collections of samples, for diverse cell types, in disease-relevant conditions. The widespread adoption of new genome editing techniques and ongoing refinement of these remarkable tools will considerably advance our ability to generate mechanistic insights into regulatory variants, but at present these lack easy scalability for higher-throughput application. It is also essential to consider the translational relevance of this work, in particular how knowledge of regulatory variants can inform drug discovery and repurposing, and how academia and pharma can work together to inform and maximize the utility of genetic studies.

## Box 1 Case studies in defining regulatory variants

***SORT1*, LDL cholesterol and myocardial infarction**

A pioneering study by Musunuru and colleagues published in 2010 [100] demonstrated how the results of a GWAS for a human disease and linked biochemical trait could be taken forward to establish mechanism and function involving regulatory variants using a combination of approaches. Myocardial infarction and plasma levels of low-density lipoprotein cholesterol (LDL-C) are strongly associated with variants at chromosome 1p13 [101]. The authors [100] fine mapped the association and defined haplotypes and LD structure through analysis of populations of African ancestry. A combination of systematic reporter gene analysis in a pathophysiologically relevant human hepatoma cell line using human bacterial artificial chromosomes spanning the 6.1 kb region containing the peak LD SNPs together with eQTL analysis established that a SNV, rs12740374, was associated with allele-specific differences in expression. eQTL analysis showed association with three genes, most notably with *SORT1* (higher expression was associated with minor allele at the transcript and protein level), and the effects were seen in liver but not subcutaneous and omental intestinal fat. The minor allele created a predicted binding site for C/EBP transcription factors, and allele-specific differences were seen using electrophoretic mobility shift assays and ChIP. Manipulating levels of C/EBP resulted in loss or gain of allelic effects on reporter gene expression and, in cells of different genotypic background, effects could be seen on SORT1 expression; human embryonic stem cells were used to show that this was specific to hepatocyte differentiation. Small interfering (siRNA) knockdown and viral overexpression studies of hepatic *Sort1* in humanized mice with different genetic backgrounds demonstrated a function for *Sort1* in altering LDL-C and very-low-density lipoprotein (VLDL) levels by modulating hepatic VLDL secretion. A genomic approach thus identified *SORT1* as a novel lipid-regulating gene and the sortilin pathway as a target for potential therapeutic intervention [100].

***FTO*,*RFX5*and obesity: effects at a distance**

Regulatory variants may modulate expression of the most proximal gene, but they can have effects at a significant distance (for example, by DNA looping or modulation of a gene network) making resolution of the functional basis of GWAS signals of association difficult [55]. Recent work on obesity-associated variants in the dioxygenase *FTO* [102] highlights this and illustrates further approaches that can be used to investigate GWAS signals and the functional significance of regulatory variants. A region spanning introns 1 and 2 of the *FTO* gene shows highly significant association with obesity by GWAS [103-105]. Following this discovery, *FTO* was found to encode an enzyme involved in control of body weight and metabolism based on evidence from FTO-deficient mice [106] and from a study of mouse overexpression phenotypes in which additional copies of the gene led to increased food intake and obesity [107]. There was not, however, evidence linking the GWAS variants or associated region with altered *FTO* expression or function. Smemo and colleagues [102] considered the wider regulatory landscape of *FTO* and mapped the regulatory interactions between genomic loci using 3C. Strikingly, their initial studies in mouse embryos revealed that the intronic GWAS locus showed physical interactions not only with the *Fto* promoter but also with the *Irx3* gene (encoding a homeodomain transcription factor gene expressed in the brain) over 500 kb away. The interaction with *Irx3* was confirmed in adult mouse brains and also human cell lines and zebrafish embryos. Data from the ENCODE project showed that the intronic *FTO* GWAS region is conserved, and its chromatin landscape suggested multiple regulatory features based on chromatin marks, accessibility and transcription factor binding. Smemo *et al.* [102] then established that the sequences have enhancer activity in relevant mouse tissues, showing that expression of *Irx3* depends on long-range elements. Strikingly, the GWAS variants associated with obesity showed association with levels of expression of *IRX3* but not of *FTO* in human brain samples. Moreover, *Irx3* knockout mice showed up to 30% reduction in body weight through loss of fat mass and increased basal metabolic rate, revealing a previously unrecognized role for IRX3 in regulating body weight. The multifaceted approach adopted by Smemo and colleagues [102] illustrates several of the approaches that can be used to define regulatory variants and the benefits of using data generated from humans and model organisms. However, the question of what the causal functional variants are and the molecular/physiological mechanisms involving IRX3 and FTO remain the subject for further work.

## Box 2 Key questions

- What are the modulated genes underlying GWAS loci?
- By what specific mechanisms do particular disease-associated regulatory variants act?
- How can we resolve regulatory variants in a disease context?
- Can epigenomic profiling of chromatin accessibility and modifications be applied to small numbers of cells?
- Are genome editing techniques amenable to throughput experiments?
- How can we use knowledge of disease association integrated with functional evidence to repurpose existing therapeutics?
- Can knowledge of disease-associated regulatory variants and modulated genes provide new drug targets for development?
- Will regulatory variants, in particular those acting in trans, provide new insights into biological pathways and networks?

## Abbreviations

3C: Chromatin conformation capture
ChIP: Chromatin immunoprecipitation
*cis*-eQTL: Local likely *cis*-acting eQTL
CRISPR: Clustered regularly interspaced short palindromic repeats
ENCODE: ENCyclopedia Of DNA Elements
eQTL: Expression quantitative trait locus
FANTOM5: Functional Annotation of the Mammalian Genome project 5 project
GWAS: Genome-wide association study
HTS: High-throughput sequencing
IFN: Interferon
LCL: Lymphoblastoid cell line
LD: Linkage disequilibrium
pQTL: Protein quantitative trait locus
QTL: Quantitative trait locus
SNV: Single-nucleotide variant
TNF: Tumor necrosis factor
*trans*-eQTL: *trans* association involving distant, likely *trans*-acting variants
